# Help seeking behavior for problematic substance uses in north-West Ethiopia

**DOI:** 10.1186/s13011-019-0202-9

**Published:** 2019-06-07

**Authors:** Habte Belete, Tesfa Mekonen, Wubalem Fekadu, Getasew Legas, Asmamaw Getnet

**Affiliations:** 1 Psychiatry Department, College of medicine and health Sciences, Bahir Dar University, Amhara region, PO Box 79, Bahir Dar, Ethiopia; 2 Psychiatry Department, College of medicine and health Sciences, Debre Tabor University, Amhara region, Debre Tabor, Ethiopia; 3 Psychiatry Department, College of medicine and health Sciences, Debre Markos University, Amhara region, Debre Markos, Ethiopia

**Keywords:** Help seeking, Problematic substance use, Urban, Ethiopia, Low-income country

## Abstract

**Background:**

Substance miss use and neuro-psychiatric conditions are a growing public health challenges, but 76 to 85% of people with those disorders in low and middle-income countries did not receive treatment. The aim of this study was to see help seeking behavior and determinant factors in residents with problematic substance uses.

**Methods:**

A total of 2400 participants had screened for problematic substance uses and 548 participants were found positive for problematic substance uses. Then, we had interviewed 548 participants for help seeking behavior by pre-tested modified General Help Seeking Questionnaire. Logistic regression with its corresponding *p*-value < 0.05; Adjusted odds ratios (AOR) and 95% confidence intervals (CI) have been used.

**Results:**

Among five hundred and forty-eight participants with problematic substance use, only one hundred and sixty-eight (30.7%) with 95% CI (27, 35%) had sought help. Age above 35 years [AOR = .47 95% CI (.25, .90)], who had common mental disorders [AOR = 4.12, 95% CI (2.7, 6.3)], who had comorbid medical condition [AOR = 3.0, 95% CI (1.7, 5.3)], and grand-families’ history of substance use [AOR = 2.18, 95% CI (1.4, 3.4)] were significantly associated with help seeking behavior.

**Conclusion:**

Help-seeking behavior was infrequent in people with problematic substance use. Advanced age was a barrier to seek help while medical illnesses, common mental disorders and history of substance use in grand families were found to enforce to seek help.

## Background

Mental, neurological and substance use disorders are common. Approximately one in four families has at least one member with a mental disorder. It also contributed for 14% of the global burden of disease. Globally, about 190 million drug users were reported and of them 40 million serious drug related illnesses or injuries were identified each year [1].

Alcohol alone contributes for 7.6 and 4% of deaths females and males respectively. It also contributes to more than 200 alcohol related diseases [2, 3]. However, 76 to 85% of people with mental, neurological and substance use disorders in low- and middle-income countries did not receive treatment for their behavioral problems [4–6].

Help-seeking is an adaptive coping process in which the individual attempts to obtain external assistance to deal with a mental health concern formally or informally. Formal help-seeking is assistance from professionals whereas informal help-seeking is assistance from informal social networks, such as friends and families [7].

Based on different circumstance help-seeking behavior for behavioral disturbance is varied a across regions and populations. Among people with mental disorders including, alcohol use disorders, only 31.7% of them had sought help. and 15.7% of sought help was from mental health providers, 8.4% from general practitioners, and 7.6% from religious/ spiritual advisors or other healers [8]. Professional help seeking behaviors among problematic drug user lesbian and bisexual women was only 41.5% [9]. Among people with alcohol use disorders 7% of them seek help from any mental health professional; 5.8% from medical health professional; 7.2% from a social support setting and 4.5% from any religious or spiritual advisor/healer [8]. Among patients who visited traditional healers for their mental health problems, 41% was visited to seek help for substance use disorders [10].

There are factors which serve as barriers to seek help for substance related behavioral disturbances [11]. From WHO report factors like, personal motivation, perception of need, lack of health insurance, internalized gender norms, and perceptions of social supports as positive, and others influence the help-seeking behaviour of adolescents’ [12]. Factors related to enrolling in problematic substance use treatment are multiple and researchers had blamed factors like lack of availability, transportation, and insurance [13]. Even though, men report higher levels of problematic substance use than women and are more likely to have psychosocial problems, but are less likely to seek help [14]. Older adults’ positive attitudes and treatment beliefs [15]; negative beliefs about the quality and effectiveness of treatment [16]; reluctance to give up the substance and to admit the need for help, and inability to afford treatment [17] were the determinant factors to use mental health services.

In Ethiopia, substance use in the community [18] and in university students has many social and academic related problems [19–21]. Despite the high prevalence of substance related problems, most people do not access professional help seeking. For instance, WHO reported that universally alcohol use disorder has a widest treatment gap (78.1%). Even though problematic substance use is remaining a major public health concern, barriers to treatment have not been well searched in developing world [22]. In Ethiopia, magnitude of problematic substance use reaches up to 22.8% in the general population [23] and health care service has been given with different modern health care settings, from holy water and traditional medicine. In Ethiopia, people with behavioral disturbance always seek traditional or/and modern health care as culturally acceptable manner. Currently, there is a growing awareness about availability of treatment options for substance misuse in Ethiopia. In one study, among lifetime khat chewer high school students, 70% of them want to stop their habit of chewing [24]. However, as per our best knowledge there is no publishing data to show prevalence and factors that associated with help seeking behaviour among people with problematic substance use in Ethiopia. Therefore, the purpose of this study was to see the level of help seeking behaviour and its contributing factor among people with problematic substance uses in urban residents.

## Methods

### Study settings

We have conducted a community based cross-sectional study in Bahir Dar town Northwest Ethiopia which is 565 k meters from Addis Ababa. The town has a total of 180,174 populations in 6 sub-cities; of these 93,014 are females. Currently, there are four hospitals, ten health centers and a number of other private health institutions (clinics, pharmacies and drug shops). In this town alcohol and khat (a green stimulant leaf) use are common among adolescents [25, 26]. Psychiatric service is delivered through formal settings (private and governmental hospitals, clinics) and informal settings (holy water and traditional medicines).

### Participants

The study was targeting on the adult residents in Bahir Dar town. Participant’s age 18 years and above were included and those participants who couldn’t communicate well for data collectors were not included. Participants who were positively screened for problematic substance use were considered for further interview.

### Sampling, selection and data collection

The sample size was determined with a single population formula by using proportion of help seeking for problematic substance uses among residents 41% [10], 4% margin of error, and 95% confidence interval. From total samples from the calculation 9 was withdrawn, 13 were unable to complete the interview and 11 refuse to continued. Finally, 548 participants were complete the interview. Since the study was two stage; we had screened a total of 2400 individuals for problematic substance use by using CAGE AID screening, until we had reach up 548 participants with problematic substance use (Khat, alcohol, tobacco and cannabis) and then assessed for help seeking. The CAGE AID screening was pre-tested for our study and has good internal consistency (@ = 0.78). Multistage sampling technique was used to select the sub-cities (three) from the total of six and to select respective administrative kebeles (the smallest administrative unit) (four) from total of seventeen. The households in the administrative kebeles were selected by simple random, and only one adult member of the house hold was selected by lottery method for the interview towards for problematic substance uses. Data was collected by degree holder nurses with interview by semi-structured questionnaire which was translated into Amharic version (local working language).

### Measurements

CAGE AID (Cut down, Annoyed, Guilty, and Eye-opener) questionnaire was used to screen for problematic substance uses. Scoring of two or greater positive answers from the four questions to social drugs (khat, alcohol, tobacco and cannabis) was considered as problematic substance uses [27]. CAGE-AID is able to address for other problematic drug uses (khat, tobacco and cannabis) in addition to screen problematic alcohol uses. Each has yes and no response which is valued one point (1) and zero (0).

Help seeking was assessed if individuals sought assistance for their substance related problems from delineated formal and informal source [7]**.** Help seeking behavior for problematic substance use was assessed by using modified General Help Seeking Questionnaire (GHSQ). It assesses whether help was sought or not and the potential sources of that help for the last 12 months. Participants were asked whether they did seek help or not for their substance use problems; and if they did seek help, they were interviewed for the source of help. GHSQ has good validity, reliability, and reliability (chronbach’s alpha = 0.83) [28] and we did a pre-test for our study and has internal consistency (chronbach’s alpha = 0.80). Social support assessed by using oslo-3 social support scale that consist three items [29]. Common mental disorder was considered when adults who score 11 or more symptoms of the 20 self-reporting questionnaire in the last one month. The self- reporting questionnaire developed to screening the presence of common mental disorders among community participants and developed by WHO [30]. Income was assessed by using relative income by leveling their own income as less than others; similar to others and better than others. Education was categorized in uneducated (who were unable to read and write); and educated (which were able to read and write). Clinical variables like, co morbid diagnosis medical illnesses were assessed by asking the participants, if they had a diagnosis of medical illnesses before the survey.

### Analysis

After the data was checked for completeness and consistency, it was coded and entered in the Epi-Data 3.1 software. Data was exported to SPSS for analysis and *p*-value less than 0.05 was declaration of a statistically significant. Bivariate and multiple logistic regression analyses had done to identify determinants of help seeking behavior for substance use related problems.

### Ethical clearance

Ethical clearance was obtained from Ethical Review committee of college of medicine and health sciences, Bahir Dar University. Formal permission letter was taken from administrative of the city and written consent was taken from the participants. All participants who were screened with a problematic substance use were referred to clinic for better screening to mental and medical illnesses. Those who were positive for screening of common mental disorders were link with psychiatric clinics.

## Results

From the total of 548, 422 (77%) of them were males. The median age of participants was 27 years and substantial number of them were living alone, 241 (44%) (Table 1). One hundred sixty-one (29.4%) had substance user family members; two hundred forty (37.2%) had substance user friends and three hundred nineteen (58.2%) had reported history of substance use among their grandparents. Two hundred three (37%) participants with a problematic substance use had found with poor social support and one hundred forty-nine (27.2%) had common mental disorders (Table 2).

**Table 1:** Socio-demographic characteristics and help seeking behavior among participants with problematic substance use (*n* = 548)

Characteristics	Help seeking behavior		Overall (%)	Chisq test(X^2^) (*p*-value)
	Yes	No		
Sex				
Male	135	287	422 (77%)	1.5(.21)
Female	33	93	126 (23%)	
Education				
Uneducated	39	62	101 (18.4%)	3.7(.05)
Educated	129	318	447 (81.6%)	
Ethnicity				
Amhara	131	304	435 (79.4%)	.3(.58)
Others^a^	37	76	113 (20.6%)	
Age				
18–24	56	106	162 (29.6%)	5.5 (.06)
25–35	91	196	287 (52.4%)	
> 35	21	78	99 (18.1%)	
Current marital status				
Married	38	105	143 (26.1%)	1.5(.22)
Unmarried	130	275	405 (73.9%)	
Relative wealth				
Lower	78	148	226 (41.2%)	2.7(.25)
Same	73	191	264 (48.2%)	
Better	17	41	58 (10.6%)	
Living circumstance				
With family	103	204	307 (56%)	2.7(.09)
Alone	65	176	241 (44%)	
Job				
Yes	78	188	266 (48.5%)	4(.51)
No	90	192	282 (51.5%)	

^a^(Oromo, Tigray, Gurage)

**Table 2:** psychosocial factors related to help seeking behavior among participants with problematic substance use (*n* = 548)

Characteristics	Help seeking behavior		Overall (%)	Chisq test (X^2^) (*p*-value)
	Yes	No		
History of substance use in grand family				
Yes	125	194	319 (41.8%)	26.1(.000)
No	43	186	229 (58.2%)	
Who had substance user friend				
Yes	78	126	204 (37.2%)	8.8(.003)
No	90	254	344 (62.8%)	
Substance use leads economic crisis				
Yes	124	269	393 (71.7%)	.5(.46)
No	44	111	155 (28.3%)	
Common mental disorders				
Yes	85	64	149 (27.2%)	67.0(.000)
No	83	316	399 (72.8%)	
Comorbid medical illnesses				
Yes	38	33	71 (13%)	20.0(.000)
No	130	347	477 (87%)	
Awareness about substance cause mental illness				
Yes	131	278	409 (74.6%)	1.4(.23)
No	37	102	139 (25.4%)	
Level of social support				
Poor	71	132	203 (37%)	3.8(.15)
Moderate	81	195	276 (50.4%)	
Good	16	53	69 (12.6%)	

### Magnitude of help seeking behavior

Only one hundred and sixty-eight (30.7%) with 95% CI (27, 35%) sought help for their substance related behavioral disturbance among total five hundred forty-eight participants with problematic substance use. Most of them were sought help from the informal source. Among these 124 (22.6%) sought help from their love; one hundred and ten (20.1%) from their friends; one hundred and three (18.8%) from their families; eighty-four (15.3%) from their relatives; and one hundred (18.2%) from religious institutes. From the formal help seekers ninety (16.4%) sought help from mental health professionals; and eighty-nine (16.2%) from general medical practitioner.

### Help-seeking for specific substance

The proportion of help-seeking behavior across different substance has a certain variation. From the total participants who has a problematic alcohol use 134/391 (34.3%) has sought help, from a problematic cigarette smoking 109/277 (39.3%) has sought help, from a problematic khat chewing 136/432 (31.5%) has sought help, and from a problematic cannabis user 63/154 (40.9%) has been sought help for their behavioral problems.

In terms on their mental health status among help seekers, 50.6% had common mental disorders, 42.3% had poor social support and 9.5% had strong social support. Among help seekers gender difference was examined and 32% male and 26.2% females were sought help.

### Multiple logistic regression analysis

During bivariate analysis advanced age, having substance user friends, comorbid medical illnesses, had grand-parents with history of substance use, and positively screened for common mental disorders were candidates for multiple logistic analysis. However, after multiple logistic regression of help seeking behavior in relation to all independent variables; advanced age, comorbid medical illnesses, had grand-parents with history of substance use, and positively screened for common mental disorders were found to be statistically significant (Table 3).

**Table 3:** Factors association with help seeking behavior for problematic substance uses

Variables	Help seeking		(Crude odd ratio) (95% CI)	AOR 95% CI	*p*-value
	Yes	No			
Age in years					
18–24	56	106	1.00	1.00	
25–35	91	196	.88 (.58, 1.32)	.89 (.57, 1.41)	.63
> 35	21	78	.51 (.28, .91)	.47 (.25, .90)^a^	.022
Common mental disorders					
Yes	85	64	5.0 (3.37, 7.58)	4.12 (2.7, 6.3)^a^	<.0001
No	83	316	1.00	1.00	
Medical illnesses					
Yes	38	33	3.1 (1.85, 5.11)	3.00 (1.69, 5.34)^a^	<.0001
No	130	347	1.00	1.00	
Grand family history of substance use					
Yes	125	194	2.8 (1.87, 4.16)	2.18 (1.42, 3.37)^a^	<.0001
No	43	186	1.00	1.00	
Substance user friend					
Yes	78	126	1.7 (1.2, 2.5)	1.43(.95, 2.16)	.087
No	90	254	1.00	1.00	

^a^ (significance at *P* value <.05) and 1.00 (reference)

## Discussion

In the current study 30.7% of the participants sought help for their substance related problems. The result is similar with the global report and a study done in Singapore 31.7% [8], but less than Los Angeles study 41.5% [9] and South African study 41% [10].

Formal help seeking behavior in this study (16.4% from mental health professionals) found similar with Singapore study (15.7% from mental health providers) [8]. However, help seeking from general medical practitioner found higher in this study (16.2%) than Singapore (8.4%). Even though many psycho-social determinants accountable for the discrepancy of magnitude of help seeking behavior, generally modern help seeking behavior looks low in developing countries. However, a high formal help-seeking behavior has been reported in this study in a developing country. This may be due to source of study population residency area was urban, and those participants may have awareness about the availability of modern health care through different medias.

Participants’ age above 35 years old was negatively associated with help seeking behavior [AOR = .47 95% CI (.25, .90)]. This contradicted with a previous study that stated older adults were seeking more mental health services than youngers [15]. Elder adults may not have a good family support to motivate them to get assistance for their behavioral disturbance. Those elder adults may not consider others’ advice or recommendations like that of younger adults to seek help due to their experience or adaptation of their behavioral problems. Participants who screened positively for common mental disorders found four times more help seeker than those who did not have common mental disorders [AOR = 4.12, 95% CI (2.7, 6.3)]. This may be due to the co-morbid effect of common mental disorders with substance related behavioral disturbance on their daily activities. The dual effect on their mental and behavioral status has become worsen and may push them to seek help. This finding agrees with studies done in America [31, 32]. Comorbid diagnosis medical illness was identified as the determinant factor for help seeking behavior for problematic substance uses. Participants with problematic substance use and had comorbid medical illnesses were three times more likely to sought help than participants who had not comorbid medical illnesses [AOR = 3.0, 95% CI (1.7, 5.3)]. This comorbidity may result perceived fear of death or other complications among participants and/or family members, which gives an alarm to sought help from others. This finding agrees with previous study in America [33].

History of substance use in grand-families had a significant association with help seeking behavior among participants with problematic substance uses. Help seeking behavior found two times more common among participants who had grand-families with history of substance use than those who hadn’t [AOR = 2.18, 95% CI (1.4, 3.4)]. The historical impact of substance related behavioral disturbance in the family member (in terms of morbidity and mortality) may has been perceived as harmful by the family members and this might motivate individuals with a problematic substance use to sought help to prevent further disabilities in the family. The impact of the substance use on their grand-family’s health may be learned in the family members and due to the uses may alert to seek help for their problem.

### Strengths and limitations

The sample size was large enough to assess all associated factors. The study had also its own limitation which must be considered in generalization. The major limitation of the study is its lack of representation of rural population. So, we recommended studies which includes rural settings.

## Conclusion and policy implication

Help seeking behavior for problematic substance use was infrequent. Help-seeking behavior was significantly associated with age, symptoms of common mental disorders and family history of substance use problems. Interventions should be done to help people with problematic substance use by targeting with those associated factors of help-seeking behavior.

## Abbreviations

CAGE: Cut down, Annoyed, Guilty, and Eye-opener
GHSQ: General Help Seeking Questionnaire
SPSS: Stasticaly Package for Social Science
USA: United State of America
WHO: World Health Organization
